# A novel senolytic drug for pulmonary fibrosis: BTSA1 targets apoptosis of senescent myofibroblasts by activating BAX

**DOI:** 10.1111/acel.14229

**Published:** 2024-06-03

**Authors:** Mengxia Shen, Jiafeng Fu, Yunna Zhang, Yanfen Chang, Xiaohong Li, Haipeng Cheng, Yujia Qiu, Min Shao, Yang Han, Yan Zhou, Ziqiang Luo

**Affiliations:** ^1^ Department of Physiology, Xiangya School of Medicine Central South University Changsha Hunan China; ^2^ Department of Pathology, The Second Xiangya Hospital Central South University Changsha China; ^3^ Hunan Key Laboratory of Organ Fibrosis Changsha China

**Keywords:** apoptosis, BAX, BTSA1, cellular senescence, idiopathic pulmonary fibrosis, myofibroblast

## Abstract

Idiopathic pulmonary fibrosis is a progressive and age‐related disease that results from impaired lung repair following injury. Targeting senescent myofibroblasts with senolytic drugs attenuates pulmonary fibrosis, revealing a detrimental role of these cells in pulmonary fibrosis. The mechanisms underlying the occurrence and persistence of senescent myofibroblasts in fibrotic lung tissue require further clarification. In this study, we demonstrated that senescent myofibroblasts are resistant to apoptosis by upregulating the proapoptotic protein BAX and antiapoptotic protein BCL‐2 and BCL‐XL, leading to BAX inactivation. We further showed that high levels of inactive BAX‐mediated minority mitochondrial outer membrane permeabilization (minority MOMP) promoted DNA damage and myofibroblasts senescence after insult by a sublethal stimulus. Intervention of minority MOMP via the inhibition of caspase activity by quinolyl‐valyl‐O‐methylaspartyl‐[2,6‐difluorophenoxy]‐methyl ketone (QVD‐OPH) or BAX knockdown significantly reduced DNA damage and ultimately delayed the progression of senescence. Moreover, the BAX activator BTSA1 selectively promoted the apoptosis of senescent myofibroblasts, as BTSA1‐activated BAX converted minority MOMP to complete MOMP while not injuring other cells with low levels of BAX. Furthermore, therapeutic activation of BAX with BTSA1 effectively reduced the number of senescent myofibroblasts in the lung tissue and alleviated both reversible and irreversible pulmonary fibrosis. These findings advance the understanding of apoptosis resistance and cellular senescence mechanisms in senescent myofibroblasts in pulmonary fibrosis and demonstrate a novel senolytic drug for pulmonary fibrosis treatment.

## Graphical abstract

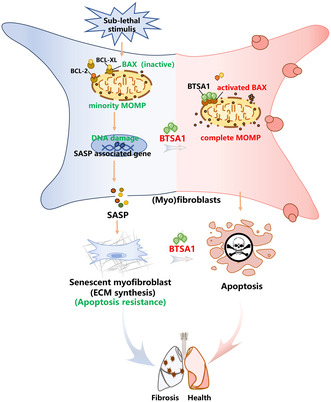

## 1 INTRODUCTION

Idiopathic pulmonary fibrosis (IPF), the most common and severe type of interstitial lung diseases, is characterized by massive deposition of extracellular matrix (ECM) and excessive remodeling of lung tissue, hardening the organs (Moss et al., 2022; Yang et al., 2023). IPF is a typical aged‐related disease (Faner et al., 2012; Guan et al., 2022; King et al., 2011). Cellular senescence is an evolutionarily conserved state characterized by permanent arrest of cell replication. Established senescence biomarkers, including increased activity of senescence‐associated‐galactosidase (SA‐β‐GAL) and cell cycle arrest proteins (p16 and p21), have been detected both in fibroblasts and alveolar epithelial cells in human IPF tissue and bleomycin (BLM)‐induced pulmonary fibrosis tissue in mice (Kunitake et al., 1998; Lomas et al., 2012; Rangarajan et al., 2022; Schafer et al., 2017).

Myofibroblasts are important effector cells during the development of pulmonary fibrosis (Kendall & Feghali‐Bostwick, 2014; Ortiz‐Zapater et al., 2022). Myofibroblasts evade apoptosis in response to pro‐survival signals from the fibrotic micro‐environment, which may ultimately lead to the senescent phenotype (Hinz & Lagares, 2020). Mitochondria play an important role in apoptosis, a process that dependent on the aggregation of BAX or BAK on the outer mitochondrial membrane, leading the mitochondrial outer membrane permeabilization (MOMP), finally causing cell death (Singh et al., 2019). Notably, only a small portion of mitochondria can undergo MOMP instead of cell death after stimulus, which is known as minority MOMP (Cao et al., 2022; Ichim et al., 2015). Mitochondrial dysfunction contributes to senescence; interestingly, mitochondrial dysfunction also promotes the localization of proapoptotic proteins in the mitochondria, which in turn promotes minority MOMP (Cao et al., 2022). However, the relationship between the minority MOMP and cellular senescence requires further investigation. Evasion of apoptosis in myofibroblasts is a hallmark of fibrotic diseases (Hinz & Lagares, 2020). Dynamic interactions of the B‐cell lymphoma (BCL‐2) family of pro and antiapoptotic proteins mediate the likelihood of whether or not they will enter apoptosis, which is referred to as the apoptotic threshold (Ashkenazi et al., 2017; Cotter, & Al‐Rubeai, M., 1995; Hinz & Lagares, 2020; Lagares et al., 2017). As the enforcers of cell death, the ability of “activators”, such as BAX and BAK, to initiate apoptosis can be counteracted by BCL‐2 family members, which contribute to survival (Singh et al., 2019; Youle & Strasser, 2008). Kushnareva et al., (2022) suggested that minority MOMP is characterized by the absence of cell death; however, whether this is related to the occurrence of apoptotic resistance in senescent cells during pulmonary fibrosis remains unclear.

Removal of the accumulated senescent myofibroblasts is a key concern in the field of pulmonary fibrosis. Apoptosis is a complex process of clearing cells in which MOMP is a switch‐like irreversible event (Czabotar et al., 2014; Lagares et al., 2017). BTSA1, a direct pharmacological BAX activator that induces the conformational transformation of BAX, effectively promotes apoptosis in acute myeloid leukemia and multitype solid tumor cell lines (Lopez et al., 2022; Reyna et al., 2017; Zhang, Zhao, et al., 2023). Although these studies have provided the evidences that BAX activation is an effective therapy for cancer, the potential of utilizing this emerging strategy for other diseases that are resistant to apoptosis has not been explored.

Here, we demonstrated that a large number of senescent myofibroblasts were present in lung tissue at the peak of pulmonary fibrosis. We found that the high expression of the proapoptotic protein BAX and antiapoptotic proteins BCL‐2, BCL‐XL in senescent myofibroblasts caused them to evade apoptosis and accumulate in the lung tissue. Significantly, we illustrated the mechanism of myofibroblast senescence; that is, the elevated expression of BAX and its inactivated state aggregate at the outer mitochondrial membrane causing minority MOMP and accelerating senescence. Significantly, we demonstrate that BAX activator, BTSA1 eliminates senescent myofibroblasts both in vivo and in vitro. In conclusion, these findings demonstrate the therapeutic potential of BTSA1 in the treatment of established pulmonary fibrosis induced by BLM by targeting senescent myofibroblast, which can be a novel senolytic drug for pulmonary fibrosis.

## 2 RESULTS

### 2.1 Biomarkers of cellular senescence increased in lung tissues of IPF patients and mice with BLM‐induced pulmonary fibrosis

Using human datasets of biomarkers in IPF (DePianto et al., 2015), we analyzed markers of cellular senescence, such as DKN1A (p21), CDKN2A (p16) and tumor protein 53 (p53) expression, in healthy and IPF lung tissues. Their expression was upregulated in patients with IPF compared to normal controls (Figure S1a; based on median‐centered log_2_ expression). These data suggest that pulmonary fibrosis is an age‐related disease, consistent with the results of previous studies (Guan et al., 2022; Schafer et al., 2017).

In the single‐dose BLM mouse model, there was a spontaneous resolution of the fibrosis beginning around day 21 following the BLM challenge. Repetitive lung injury with BLM produces substantial architectural distortion in the lungs and fibrosis that does not spontaneously resolve, which more closely resembles IPF than lung fibrosis induced by the BLM single intratracheal instillation (Degryse et al., 2010; Song et al., 2022). Based on hematoxylin–eosin and trichrome blue collagen staining, the development of fibrosis in lung tissue peaked approximately day 21 post instillation (d.p.i.) in the reversible model, with extensive alveolar wall thickening, fibrotic foci, and collagen deposition in the lung tissues. The disruption of lung structure and the accumulation of collagen in the lung tissues of mice injected with a single dose were significantly attenuated by 35 d.p.i., whereas fibrotic features persisted in the lung tissues of mice subjected to repetitive intranasal BLM instillation at 35 d.p.i. (Figure S1c,d). The Ashcroft score, which is used to grade pulmonary fibrosis, also demonstrates this phenomenon (Figure S1f). Next, we determined the number of senescent myofibroblasts in these two models, indicated by α‐smooth muscle actin (α‐SMA) and p21, as biomarkers of myofibroblasts and cellular senescence respectively. Our results showed that the number of α‐SMA (red) and p21 (green) double positive cells peaked at 21 d.p.i., and then decreased with fibrosis resolution at 28 d.p.i. in the reversible model. However, senescent myofibroblasts were observed in fibrotic foci continuously at both 28 and 35 d.p.i. when pulmonary fibrosis was induced by repetitive BLM challenge (Figure S1e,h). Collectively, these data provide evidence that increased numbers of senescent myofibroblasts in lung fibrosis are associated with an enhanced fibrotic response.

### 2.2 Limited mitochondrial permeabilization occurs in senescent myofibroblasts

**FIGURE 1:**
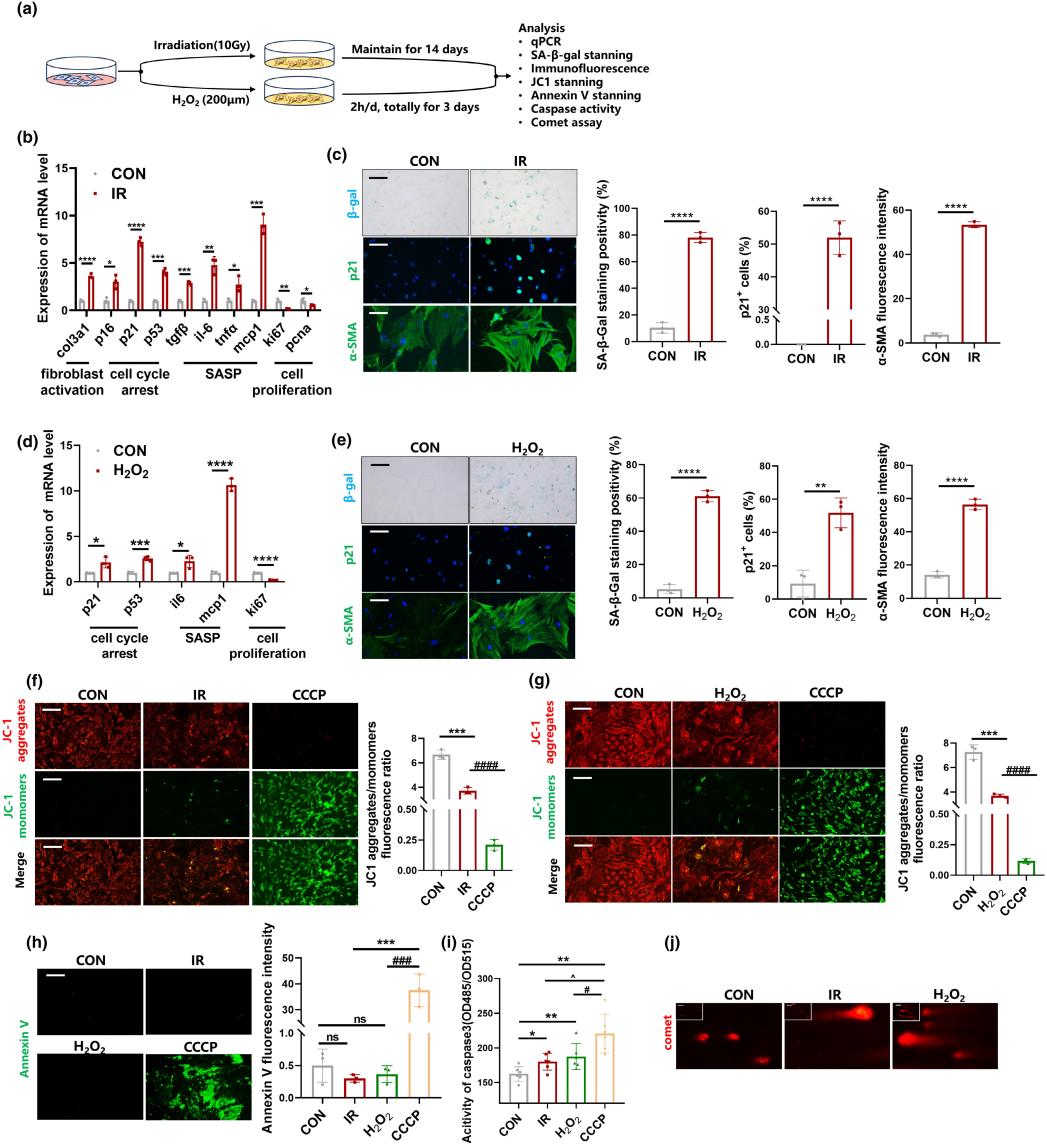
Limited mitochondrial permeabilization occurs in senescent myofibroblasts. (a) Schematic for senescent myofibroblasts induced by IR and H_2_O_2_. (b) The mRNA level of Col3a1, p16, p21, p53, tgfβ, il6, tnfα, mcp1, ki67, pcna in IR‐induced senescent myofibroblast detected by qPCR. Graph represents mean ± SEM (*n* = 3); *p < 0.05, **p < 0.01, ***p < 0.001, ****p < 0.0001. (c, e) SA‐β‐gal staining (scale bar = 100 μm), immunofluorescent staining of α‐SMA and p21 (scale bar = 50 μm). Graph represents mean ± SEM (*n* = 3); **p < 0.01, ****p < 0.0001. (d) The mRNA level of il6, mcp1, p21, p53, ki67 in H_2_O_2_‐induced senescent myofibroblast detected by qPCR. Graph represents mean ± SEM (*n* = 3); *p < 0.05, ***p < 0.001, ****p < 0.0001. (f, g) JC‐1 staining in senescent myofibroblast, scale bar = 100 μm. Graph represents mean ± SEM (*n* = 3); ***p < 0.001, ^####^ *p* < 0.0001. (h). Annexin V staining in senescent myofibroblast, scale bar = 100 μm. Graph represents mean ± SEM (*n* = 3); ***p < 0.001, ^###^ *p* < 0.001. (i) The activity of caspase 3 in senescent myofibroblasts. Graph represents mean ± SEM (*n* = 3); *p < 0.05, **p < 0.01, ^#^ *p* < 0.05, ^*p* < 0.05. (j) Comet assay in senescent myofibroblast.

To clarify the mechanism of cellular senescence of myofibroblasts, primary mouse fibroblasts were extracted and purified to the third and sixth generation. The primary mouse lung fibroblasts were then subjected to 10 Gy irradiation (IR) or 200 μm hydrogen peroxide H_2_O_2_ for three times to construct a senescent myofibroblast model in vitro (Figure 1a). qPCR assay showed that cell cycle block markers, including p16, p21 and p53, as well as senescence‐associated secretory phenotype (SASP) secreta group, including tgf‐β, il6, tnf‐α, and mcp1, were elevated in fibroblasts after IR exposure (Figure 1b). Meanwhile, IR significantly decreased the levels of cell proliferation markers (ki67 and pcna) in fibroblasts (Figure 1b). Our results showed the same trends in the level of cell cycle blockade markers, SASP secreta group and cell proliferation markers after H_2_O_2_ stimulation (Figure 1d). Moreover, we found that SA‐β‐Gal activity was significantly elevated after exposure to IR and H_2_O_2_ (Figure 1c,e). IR or H_2_O_2_ stimulation also increased the level of α‐SMA (Figure 1c,e) and the mRNA level of col3 (Figure 1b,d) in fibroblasts, which indicating the activation of fibroblasts. Collectively, these data confirm that fibroblasts can be successfully transformed into senescent myofibroblasts after IR and H_2_O_2_ stimulation, confirming the successful construction of cellular models in vitro.

**FIGURE 2:**
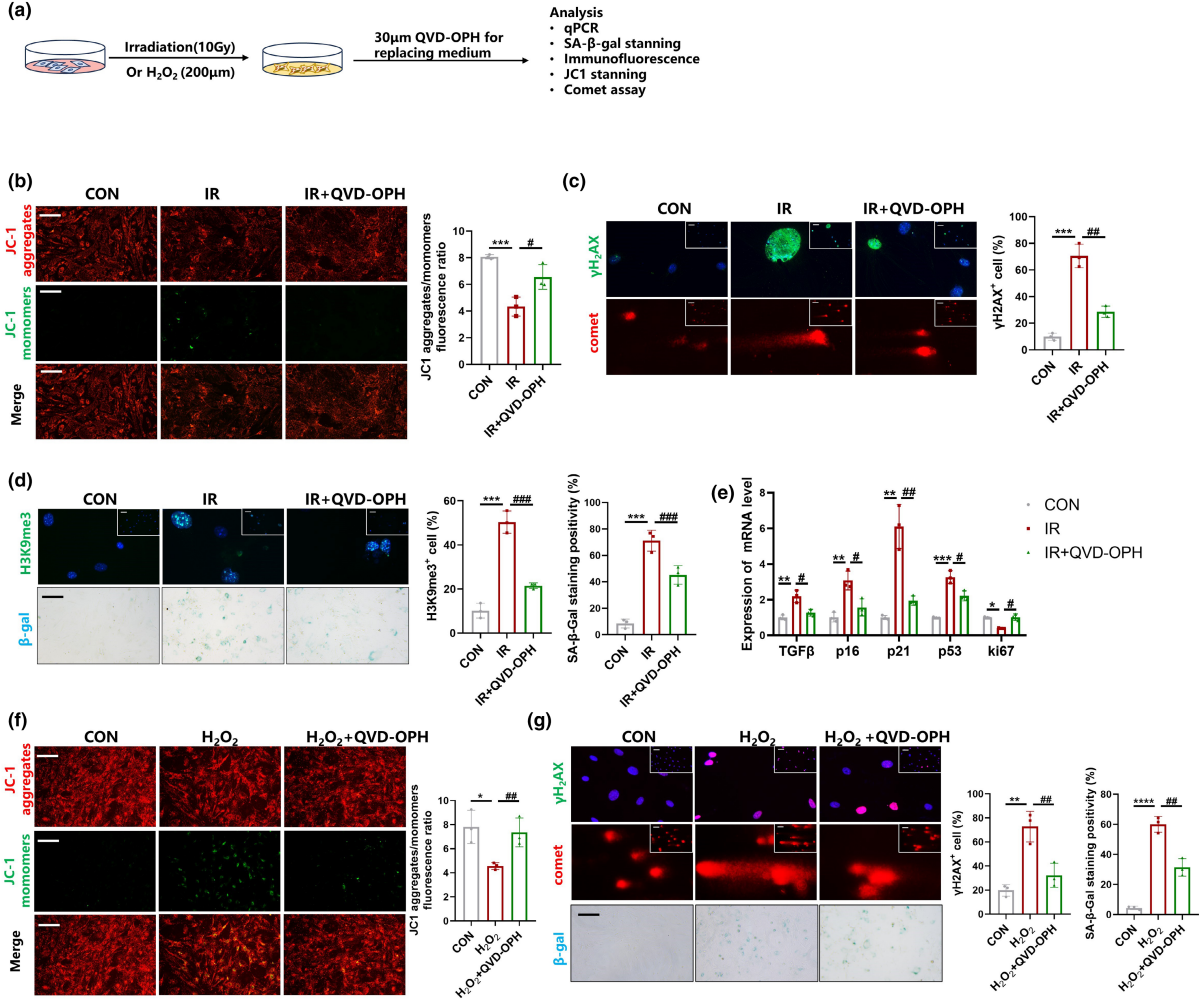
Minority MOMP induced by sublethal stimuli promoted cellular senescence in fibroblasts. (a) Schematic for senescent myofibroblasts treated with QVD‐OPH. (b, f) JC‐1 staining in senescent myofibroblast, scale bar = 100 μm. Graph represents mean ± SEM (*n* = 3); *p < 0.05, ***p < 0.001, ^#^ *p* < 0.05, ^##^ *p* < 0.01. (c) Immunofluorescent staining of γH_2_AX and comet assay (scale bar = 100 μm) in senescent myofibroblast. Graph represents mean ± SEM (*n* = 3); ***p < 0.001, ^##^ *p* < 0.01. (d) Immunofluorescent staining of H3K9me3 and SA‐β‐gal staining in senescent myofibroblast, scale bar = 100 μm. Graph represents mean ± SEM (*n* = 3); ***p < 0.001, ^###^ *p* < 0.001. (e) The mRNA level of tgfβ, p16, p21, p53, ki67 in senescent myofibroblast detected by qPCR. Graph represents mean ± SEM (*n* = 3); *p < 0.05, **p < 0.01, ***p < 0.001, #*p* < 0.05, ##*p* < 0.01. (g) Immunofluorescent staining of γH_2_AX, comet assay, SA‐β‐gal staining (scale bar = 100 μm) in senescent myofibroblast. Graph represents mean ± SEM (*n* = 3); **p < 0.01, ****p < 0.0001, ##*p* < 0.01.

Carbonyl cyanide m‐chlorophenyl hydrazone (CCCP) is an inhibitor of oxidative phosphorylation that induces reactive oxygen species (ROS)‐mediated MOMP and cell death, which was used as a positive control for minority MOMP (Cao et al., 2022). Consequently, compared to normal fibroblasts, CCCP induced a significant decrease in mitochondrial membrane potential (mΔψ) as evidenced by a decrease in red fluorescence and an increase in green fluorescence; however, the mΔψ decreased slightly in senescent myofibroblasts induced by IR and H_2_O_2_ (Figure 1f,g). Similarly, annexin V^+^ apoptotic cells were almost absent in senescent myofibroblasts, whereas apoptosis was evident after CCCP stimulation according to annexin V staining (Figure 1h). Caspases are the central components of the apoptotic machinery (Shi, 2002). The caspase activity of fibroblasts treated with IR and H_2_O_2_ showed an upward trend, although the magnitude of the elevation was much lower than that in the CCCP‐treated group (Figure 1i), indicating limited caspase activation in senescent myofibroblasts. Furthermore, the comet assay revealed that IR and H_2_O_2_ caused DNA damage (Figure 1j), which is an important feature of minority MOMP. In general, these data demonstrate that minority MOMP occurs in senescent myofibroblasts in response to sublethal stimuli (IR or H_2_O_2_) without causing cell death.

### 2.3 Minority MOMP induced by sublethal stimuli promoted cellular senescence in fibroblasts

Quinolyl‐valyl‐O‐methylaspartyl‐ [2,6‐difluorophenoxy]‐methyl ketone (QVD‐OPH), is a broad‐spectrum caspase inhibitor with potent antiapoptotic properties that inhibit minority MOMP by suppressing the caspase activity (Caserta et al., 2003). To understand the mechanism of senescence in fibroblasts and relationship between minority MOMP and senescence, we examined the progression of cellular senescence occurring in the presence or absence of QVD‐OPH (Figure 2a). JC1 staining showed that QVD‐OPH effectively alleviated the reduction in mΔψ that was induced by IR or H_2_O_2_ (Figure 2b,f). DNA damage response is a feature of cellular senescence that is induced by dysfunctional telomeres as well as DNA double‐strand breaks elsewhere in the genome. As a DNA double‐strand breaks marker, γH2AX foci are present in various senescent cells (d'Adda di Fagagna, 2008). Immunofluorescence and comet assay results suggested that QVD‐OPH can alleviate DNA damage caused by IR or H_2_O_2_ (Figure 2c,g). In addition, alterations in the chromatin structure are known to contribute to cellular senescence. The characteristic heterochromatin structures formed in senescent cells are called senescence‐associated heterochromatin foci, and can be reflected by the detection of tri‐methyl‐histone H3 (Lys9) (H3K9Me3) (Narita et al., 2003). Immunofluorescence results revealed that QVD‐OPH reduced the protein level of IR‐induced H3K9Me3, and the percentage of SA‐β‐gal positive cells (Figure 2d,g). Furthermore, QVD‐OPH decreased the mRNA leves of tgfβ, p16, p21, p53, and increased the level of ki67 in IR‐stimulated fibroblasts (Figure 2e). Taken together, these finding suggest that QVD‐OPH, which can inhibit minority MOMP by inhibiting caspases, could impede the cellular senescence progression.

### 2.4 Incomplete activation of BAX contributes to minority MOMP in senescent myofibroblasts

**FIGURE 3:**
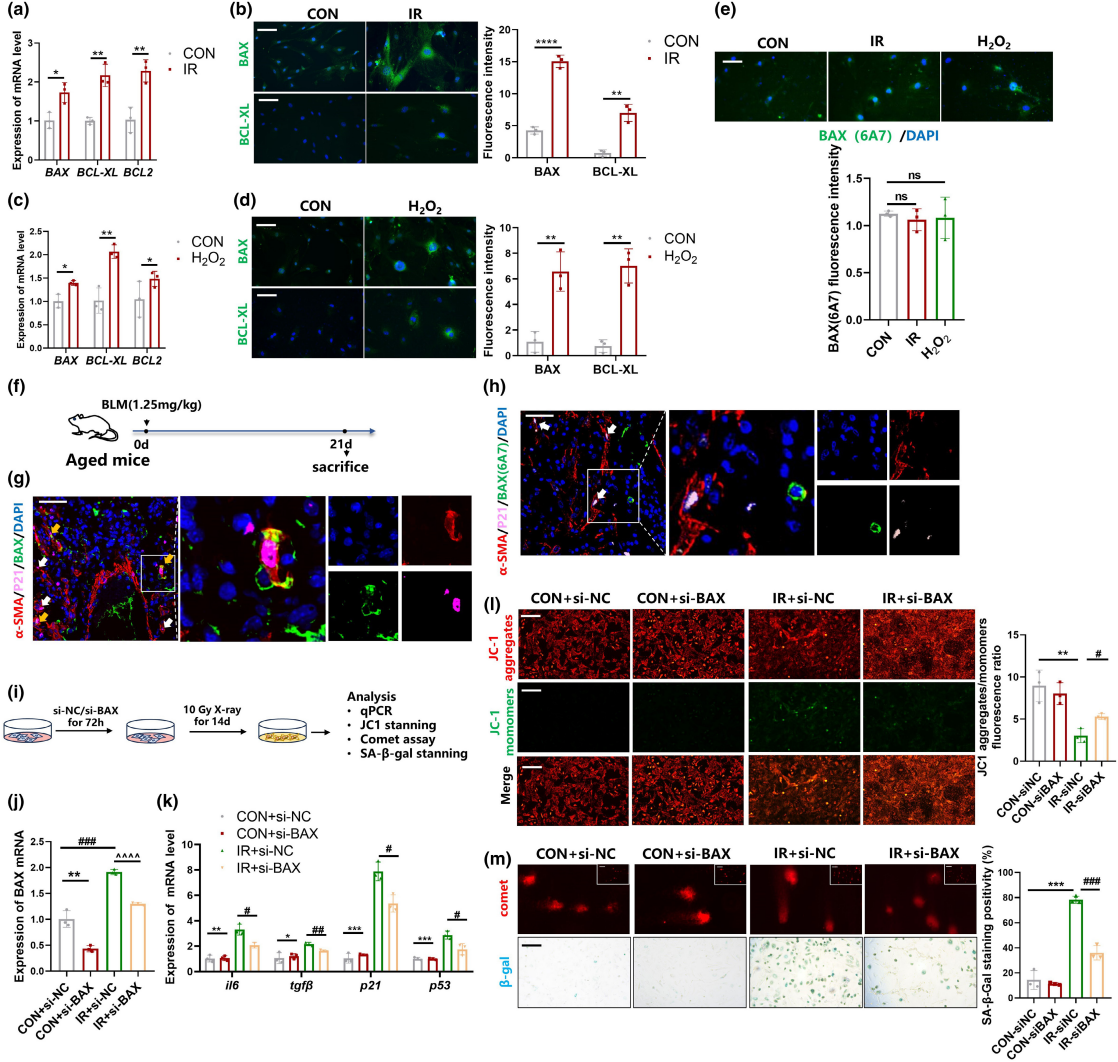
Incomplete activation of BAX promotes minority MOMP in senescent myofibroblast. (a, c) The mRNA level of bax, bcl‐xl, bcl2 in senescent myofibroblast detected by qPCR. Graph represents mean ± SEM (*n* = 3); *p < 0.05, **p < 0.01. (b, d) Immunofluorescent staining of BAX, BCL‐XL in senescent myofibroblast, scale bar = 100 μm. Graph represents mean ± SEM (*n* = 3); **p < 0.01, ****p < 0.0001. (e) Immunofluorescent staining of BAX (6A7) in senescent myofibroblast, scale bar = 100 μm. Graph represents mean ± SEM (*n* = 3). (f) Schematic for single‐dose BLM‐induced irreversible fibrosis model in aged mice. (g). Immunofluorescent staining of α‐SMA (Red) and p21 (pink) BAX (Green) in lung sections of BLM mice. White arrows point to senescent myofibroblasts, yellow arrows point to triple‐positive cells. (h) Immunofluorescent staining of α‐SMA (Red) and p21 (pink) BAX6A7 (Green) in lung sections of BLM mice. White arrows point to senescent myofibroblasts. (i) Schematic for fibroblasts transfected with si‐BAX and induced by IR into senescent myofibroblasts. (j) The mRNA level of bax detected by qPCR. Graph represents mean ± SEM (*n* = 3); **p < 0.01, ^##^ *p* < 0.01, ^^^^*p* < 0.0001. (k) The mRNA level of il6, tgfβ, p21, p53 detected by qPCR. Graph represents mean ± SEM (*n* = 3); *p < 0.05, **p < 0.01, ***p < 0.001, #*p* < 0.05, ^##^ *p* < 0.01. (l) JC‐1 staining in senescent myofibroblast, scale bar = 100 μm. Graph represents mean ± SEM (*n* = 3); **p < 0.01, #*p* < 0.05. (m) Comet assay and SA‐β‐gal staining (scale bar = 100 μm) in senescent myofibroblast. Graph represents mean ± SEM (*n* = 3); ***p < 0.001, ^###^ *p* < 0.001.

BCL‐2 protein family play a vital role in apoptosis. Lagares et al., (2017) defined “mitochondrial priming” as a measure of the proximity of mitochondria to the threshold of apoptosis, thus the dynamic balance between antiapoptotic protein and proapoptotic proteins determines whether a cell survives or undergoes apoptosis. qPCR and immunofluorescence showed that compared to the control, the mRNA and protein levels of the proapoptotic protein BAX and antiapoptotic BCL‐XL were upregulated after exposure to IR and H_2_O_2_ (Figure 3a,c), compared to the control. The mRNA level of BCL‐2 also increased after IR and H_2_O_2_ exposure (Figure 3b,d). However, there was no significant change in BAX activity as indicated by immunofluorescence for BAX6A7 in senescent myofibroblasts induced by IR and H_2_O_2_ (Figure 3e). Meanwhile, to determine whether apoptosis resistance also exists in senescent myofibroblasts in vivo, irreversible lung fibrosis was induced by tracheal injection of BLM into 20‐month‐old mice (Figure 3f). We found that α‐SMA^+^ (red) p21^+^(pink) senescent myofibroblasts expressed high levels of BAX (green) in lung fibrosis tissue (Figure 3g), but BAX6A7 (green) was almost absent in α‐SMA^+^(red) p21^+^(pink) senescent myofibroblasts (Figure 3h). These results indicate that senescent myofibroblasts in fibrotic lung tissue have high levels of BAX with low or no activity.

To investigate the effect of BAX on minority MOMP and the senescence process, we knocked down the expression of BAX three days prior to IR (Figure 3i,j). qPCR results showed that the expression of il6, tgfβ, p21, p53 induced by IR could be reversed by knocking down BAX (Figure 3k). Knockdown of BAX significantly prevented the decrease in mΔψ in the IR‐induced senescent myofibroblasts (Figure 3l). Additionally, knockdown of BAX reduced IR‐induced cellular DNA damage and arrested the senescence process as determined by comet assay and SA‐β‐Gal staining (Figure 3m). Taken together, our findings reveal that senescent myofibroblasts with have high levels of BAX but with low activity to induce minority MOMP, can survive after sublethal stimuli, and that knockdown of BAX can prevent cellular senescence by suppressing minority MOMP.

### 2.5 BTSA1‐induced BAX activation promotes apoptosis in senescent myofibroblasts

**FIGURE 4:**
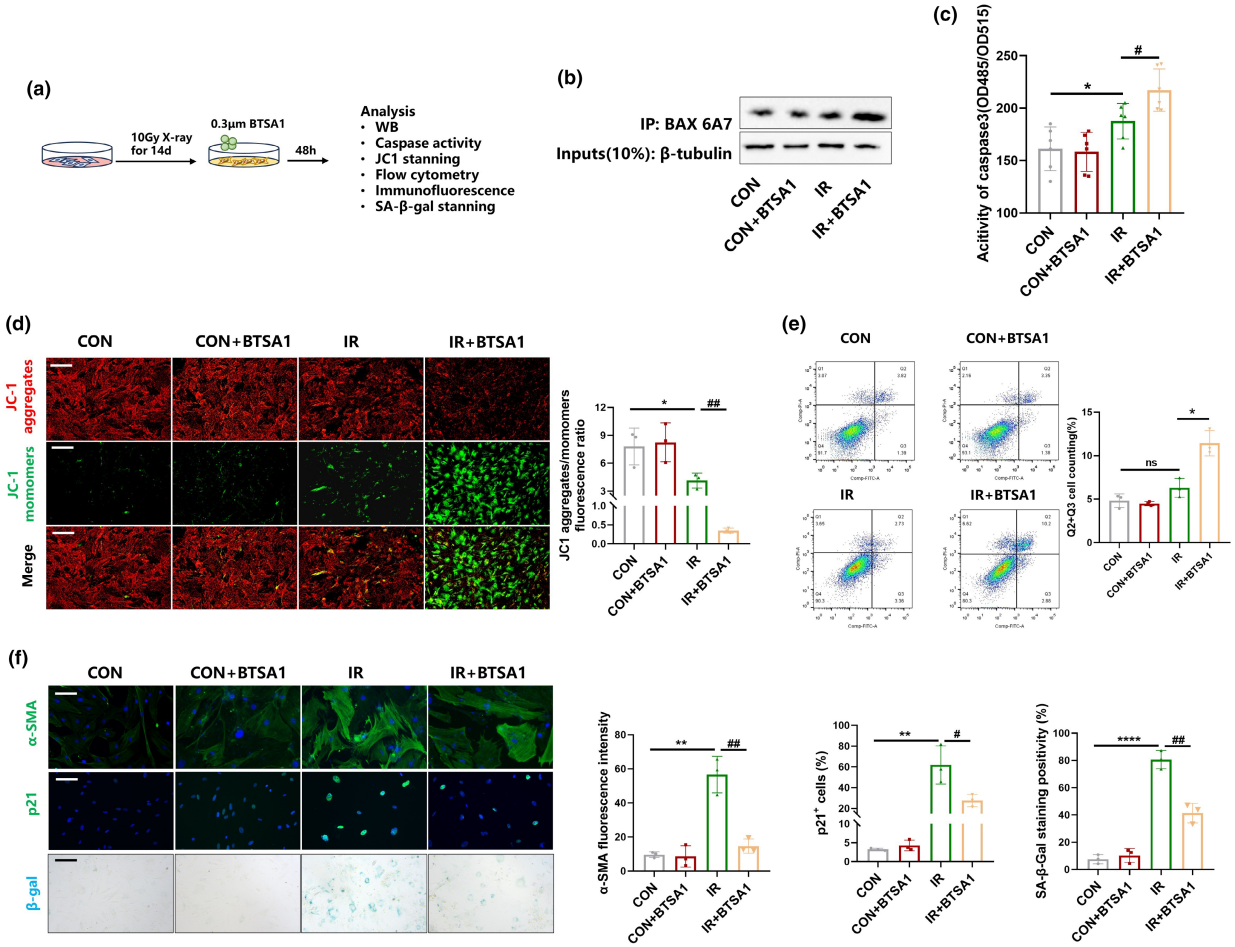
BTSA1‐induced BAX activation promotes apoptosis in senescent myofibroblast. (a) Schematic for senescent myofibroblasts treated with BTSA1. (b) The activity of BAX in senescent myofibroblasts detected by coimmunoprecipitation assay. (c) The activity of caspase 3 in senescent myofibroblast. Graph represents mean ± SEM (*n* = 3); *p < 0.05, #*p* < 0.05. (d) Cells were stained with annexin V and PI and analyzed by FACS. Cells positive for annexin V staining were counted as apoptotic cells. Graph represents mean ± SEM (*n* = 3); *p < 0.05, ^##^ *p* < 0.01. (e) JC‐1 staining in senescent myofibroblast, scale bar = 100 μm. Graph represents mean ± SEM (*n* = 3); *p < 0.05. (f) Immunofluorescent staining of α‐SMA and p21 (scale bar = 50 μm), SA‐β‐gal staining in senescent myofibroblast (scale bar = 100 μm). Graph represents mean ± SEM (*n* = 3); **p < 0.01, ****p < 0.0001, ^#^ *p* < 0.05, ^##^ *p* < 0.01.

Minority MOMP, a cellular self‐protection mechanism following sublethal stimuli, can be transformed into complete MOMP in response to proapoptotic drugs, thereby inducing apoptosis (Xu et al., 2020). To investigate the effect of BAX activation on the transformation of minority MOMP into complete MOMP, senescent myofibroblasts were treated with BTSA1, the specific activator for BAX (Figure 4a). BAX 6A7 levels were determined by immunoprecipitation and the results demonstrated that BTSA1 effectively activates BAX in IR‐induced senescent myofibroblasts (Figure 4b). Importantly, BTSA1 significantly increased the activity of caspase system and reduced the mΔψ in IR‐induced senescent myofibroblasts (Figure 4c,d). Furthermore, flow cytometry results showed that BTSA1 effectively promoted the apoptosis of senescent myofibroblasts, as BTSA1‐activated BAX converted minority MOMP to complete MOMP (Figure 4e). Moreover, the number of senescent myofibroblasts decreased with BTSA1 administration as indicated by SA‐β‐Gal staining (Figure 4f). Thus, our data indicate that BTSA1 specifically eliminates senescent myofibroblasts by promoting its apoptosis by activating the high level of low‐activity BAX directly in senescent cells.

### 2.6 BTSA1 alleviates pulmonary fibrosis both in young mice with reversible fibrosis and aged mice with irreversible fibrosis

Given the capacity of BTSA1 to eliminate senescent myofibroblasts in vitro, we next examined whether this drug could be utilized to intervene against pulmonary fibrosis pathologies in vivo.

**FIGURE 5:**
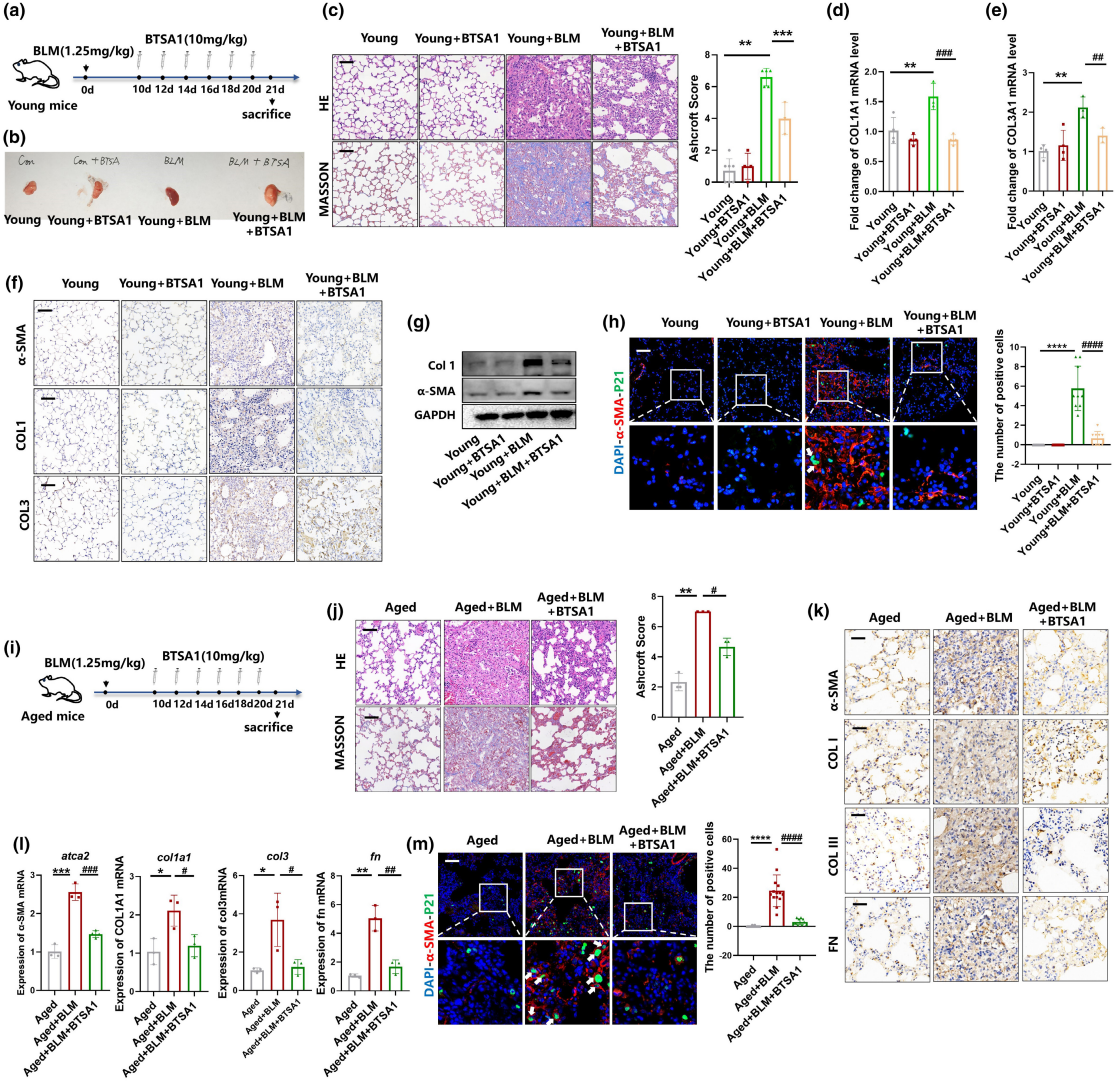
BTSA1 alleviates pulmonary fibrosis both in young mice with reversible fibrosis and aged mice with irreversible fibrosis. (a) Schematic for single‐dose BLM‐induced reversible fibrosis model in young mice. (b) Morphology of the lung samples. (c) H&E and Masson staining of lung sections in young fibrotic lung tissue of mice, scale bar = 25 μm. Graph represents mean ± SEM (*n* = 3); **p < 0.01, ***p < 0.001. (d, e) The mRNA level of COL1, COL3 in lung tissue detected by qPCR. Graph represents mean ± SEM (*n* = 3); **p < 0.01, ^###^ *p* < 0.001. (f) Immunohistochemistry staining of α‐SMA, COL1, COL3 in fibrotic lung tissue of young fibrotic mice, scale bar = 25 μm. (g) The content of COL1 and α‐SMA in lung tissue detected by western blotting. β‐tubulin were used as the loading control. Graph represents mean ± SEM (*n* = 3); **p < 0.01, ^##^ *p* < 0.01. (h) Immunofluorescent double‐staining of α‐SMA (Red) and p21 (Green) of lung sections of young fibrotic mice. White arrows point to senescent myofibroblasts. Graph represents mean ± SEM (*n* = 3); ****p < 0.0001, ^####^ *p* < 0.0001. (i) Schematic for single‐dose BLM‐induced irreversible fibrosis model in aged mice. (j) H&E and Masson staining of lung sections in aged fibrotic lung tissue of mice, scale bar = 25 μm. Graph represents mean ± SEM (*n* = 3); **p < 0.01, #*p* < 0.05. (k) The mRNA level of α‐SMA, COL1, COL3, FN in lung tissue detected by qPCR. (l) Immunohistochemistry staining of α‐SMA, COL1, COL3 and FN in fibrotic lung tissue of aged fibrotic mice, scale bar = 25 μm. Graph represents mean ± SEM (*n* = 3); *p < 0.05, **p < 0.01, ***p < 0.001, ^#^ *p* < 0.05, ^##^ *p* < 0.01, ^###^ *p* < 0.001. (m) Immunofluorescent double‐staining of α‐SMA (Red) and p21 (Green) of lung sections of aged fibrotic mice. White arrows point to senescent myofibroblasts. Graph represents mean ± SEM (*n* = 3); ****p < 0.0001, ^####^ *p* < 0.0001.

There is a strong association between IPF and age. BLM‐induced pulmonary fibrosis in young mice is characterized by self‐regression, however, the capacity for fibrosis resolution in aged mice is markedly impaired and is similar to IPF (Hecker et al., 2014). To confirm the effect of BTSA1, we constructed a reversible lung fibrosis model by tracheal injection of BLM into young mice and treated them with BTSA1 starting at 10 d.p.i. The same treatment was used to construct an irreversible lung fibrosis model in 20‐month‐old aged mice (Figure 5a,i). Visible morphological changes in fibrotic induction, such as pulmonary injury and edema, were observed in BLM group compared to those in the control group. However, lung injuries induced by BLM were significantly ameliorated in BTSA1‐treated mice (Figure 5b). H&E staining showed alveolar septum thickening and collapse in the lung tissue of the BLM group; treatment with BTSA1 significantly improved BLM‐induced thickening of alveolar septum and alveolar collapse. Masson staining showed that BTSA1 significantly reduced collagen deposition (blue staining) in lung tissue after BLM. Moreover, the Ashcroft score significantly increased after the BLM challenge and decreased after the treatment of BTSA1 (Figure 5c,j). qPCR, western blotting and immunohistochemical staining for evaluation of α‐SMA, and ECM components, like COL1, COL3, confirmed that the increased protein levels of α‐SMA in the lung tissue of BLM‐injured mice were significantly attenuated by BTSA1 (Figure 5d–g,k,l). In addition, BLM stimulation augmented the number of senescent myofibroblasts, indicated by α‐SMA (red) and p21 (green) double staining. After BTSA1 treatment, the number of α‐SMA^+^p21^+^ senescent myofibroblasts decreased (Figure 5f,m). Thus, the data from both young and old mice showed that BTSA1 can alleviates BLM‐induced pulmonary fibrosis and promoted fibrosis resolution. Furthermore, it is possible for BTSA1 to be a potential novel senolytic drug to treat pulmonary fibrosis in clinical by eliminating senescent myofibroblasts.

## 3 DISCUSSION

**FIGURE 6:**
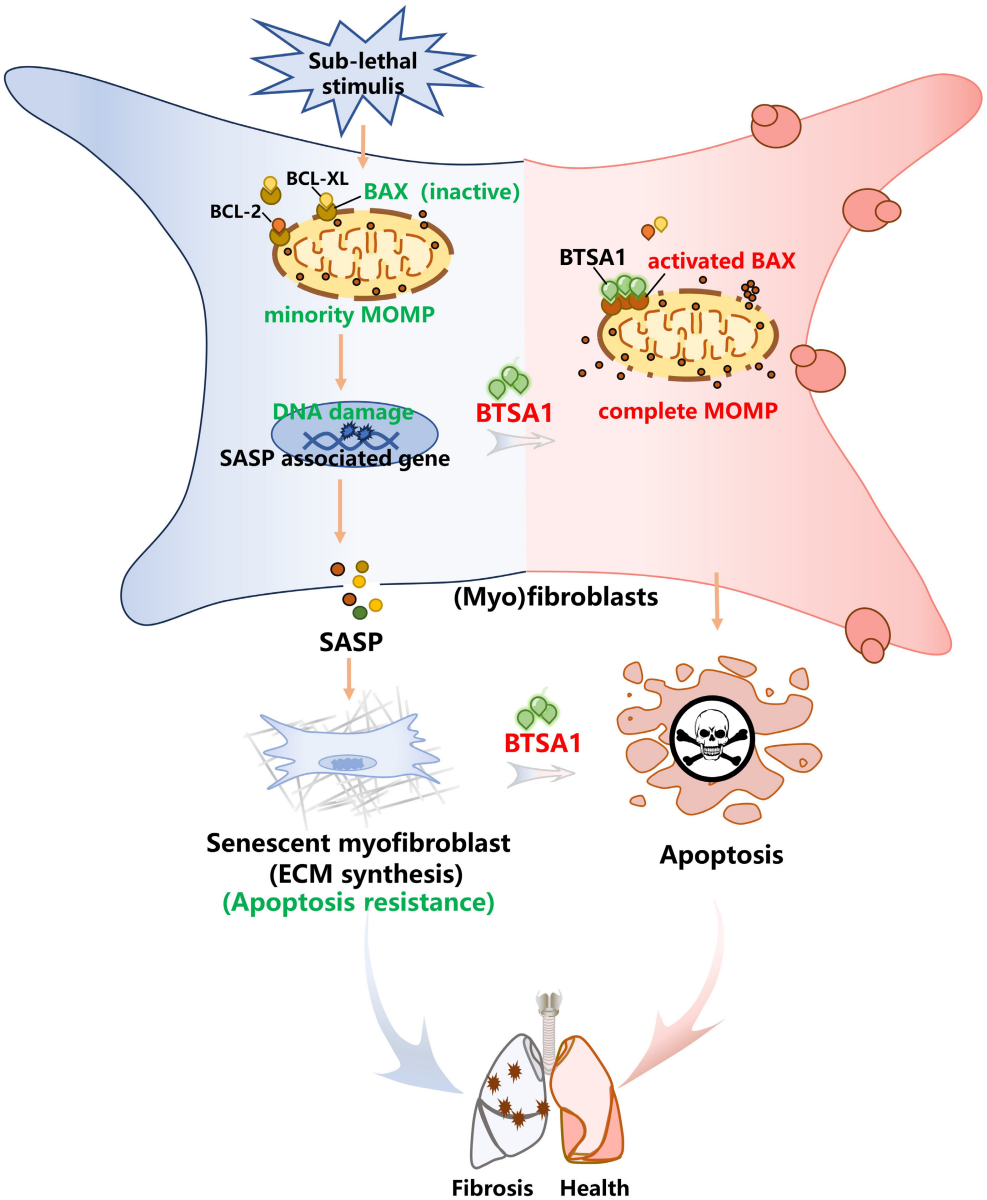
Schematic model of the anti‐fibrotic mechanism of BAX activation by the senolytic drug BTSA1.

In this study, we found that senescence in myofibroblasts may be related to BAX‐mediated minority MOMP and DNA damage. Cellular senescence induced by IR and H_2_O_2_ in myofibroblasts can be prevented by knocking down the expression of BAX or blocking minority MOMP with QVD‐OPH, which is specifically manifested as recovery of mitochondrial membrane potential, decreased levels of DNA damage and SASP factor, decreased number of SA‐β‐gal positive cells. In addition, our results showed that senescent myofibroblasts had apoptotic resistance with high levels of BAX but with low BAX activity because of high levels of anti‐apoptosis agents. Activation of BAX with BTSA1 could selectively eliminated senescent myofibroblasts in vitro and in vivo by converting minority MOMP to complete MOMP to promote apoptosis, thereby alleviating pulmonary fibrosis and blocking the continuous progression of pulmonary fibrosis in vivo (Figure 6).

It has been unquestionably that the BLM‐induced pulmonary fibrosis mouse model has been extensively used to study pulmonary fibrosis and improve our understanding of its pathogenesis. In fact, single‐dose BLM‐induced lung fibrosis spontaneously resolves around day 28 following the BLM stimulus, fails to present the progressive and irreversible characteristics of IPF in patient (Moore & Hogaboam, 2008; Zia et al., 1992). Earlier studies demonstrated that the extent and severity of lung lesions continue to increase after three or more BLM stimulations. Three consecutive BLM stimulations per week can lead to chronic, persistent pulmonary fibrosis (Kendall & Feghali‐Bostwick, 2014). Young mice with repetitive intranasal BLM instillation are characterized by persistent progressive pulmonary fibrosis, including loss of alveolar epithelial cells in fibrotic areas, increased collagen deposition, fibroblast accumulation and honeycomb cysts and resembles IPF more closely (Degryse et al., 2010; Redente et al., 2021; Ye et al., 2023). Our results suggest that in the reversible model, pulmonary fibrosis regresses spontaneously at 35 d.p.i., mainly in the form of lung tissue remodeling and reduced collagen deposition, while the lung fibrosis persisted at 35 d.p.i. in irreversible model. What is the most important is that senescent myofibroblasts persist in irreversible lung injury but decrease with fibrosis resolution at 28 d.p.i. in the reversible model, implying that senescent myofibroblasts may be associated with the progressive character of IPF.

Myofibroblasts are the key effectors of scarring and fibrosis, and respond to excessive composition, deposition and remodeling of the ECM (Merkt et al., 2021). Previous studies have shown that myofibroblasts evade apoptosis in response to pro‐survival biomechanics and growth factor signals from the fibrotic microenvironment, may ultimately leading to the acquisition of a senescent phenotype (Hinz & Lagares, 2020; Horowitz et al., 2012). Evasion of apoptosis through the dysregulation of specific BCL‐2 family genes is a recurrent event in disease states. We found that existence of apoptosis resistance in senescent myofibroblasts in pulmonary fibrosis is dependent on “mitochondrial priming”, which may be caused by the simultaneous high expression of BAX and antiapoptotic proteins (BCL‐XL and BCL‐2). Thus, the inactivation of BAX caused by high levels of anti‐apoptosis in senescent myofibroblasts is an important mechanism of apoptosis evasion, resulting in the persistence in lung fibrotic tissue.

MOMP is a switch‐like event during apoptosis and is dependent on the accumulation of BAX and BAX on mitochondria (Nechushtan et al., 2001). However, the inactive BAX mitochondrial location would promote minority MOMP with limited caspase activation, which is insufficient to trigger cell death, and minority MOMP‐induced caspase activity could cause DNA damage and mitochondrial dysfunction, which are features of cellular senescence (Hernandez‐Segura et al., 2018). Our data revealed that DNA damage caused by minority MOMP, which is mediated by high levels of inactive BAX, is involved in the process of senescence in myofibroblasts. Intervention of minority MOMP via inhibition of caspase activity by QVD‐OPH or knocking down of BAX restored the decreased mΔψ, reducing DNA damage and ultimately delaying senescence. Interestingly, the latest research published in NATURE (Victorelli et al., 2023) also showed that mitochondrial DNA released by minority MOMP can drive SASP by activating cGAS–STING signaling pathways, and that inhibition of minority MOMP by BAI1, a small‐molecule BAX inhibitor, reduces inflammation and cellular senescence. This important study finding, as well as the results from current study, simultaneously show that minority MOMP induced by sublethal stimuli contributes to cellular senescence, whereas inhibition of minority MOMP could prevent the occurrence of senescence. In addition, BAX knockdown reversed the IR‐induced elevation of il6, tgfβ, p21, and p53 expression, ameliorated the mΔψ, reduced the DNA damage, and decreased the number of SA‐β‐gal‐positive cells, suggesting that BAX‐mediated minority MOMP is an important mechanism senescence in myofibroblasts and a key mechanism for apoptosis resistance in senescent myofibroblasts.

Senolytic therapies are considered as a promising approach to treat age‐related diseases (Zhou & Lagares, 2021). However, current drug therapies are accompanied by adverse side effects. In a xenograft mouse model of multiple myeloma and BCL‐2, ABT‐263 exhibited increased toxicity when mice were treated with the BCL‐2, BCL‐XL, and BCL‐W inhibitor ABT‐263 (Tse et al., 2008). Similarly, in patients treated daily with ABT‐263, patients experience transient thrombocytopenia and neutropenia (Rudin et al., 2012). Another BCL‐2, BCL‐XL and BCL‐W inhibitor, ABT‐737 has been shown to activate minority MOMP, thereby promoting tumorigenesis (Ichim et al., 2015). When larger doses of two BCL‐XL inhibitors, A1331852 and A1155463, were co‐administered to proliferating cells, cellular activity was inhibited (Zhu et al., 2017). Therefore, it is important to find a novel senolytic drug to treat the disease while reducing the toxic side effects. It was found that when BTSA1 was used in combination with ABT‐263, it was possible to use a lower dose of ABT‐263 which is generally safe in terms of tissue and blood cell counts. It is suggested that BTSA1 may be a novel senolytic drug with less toxic side effects (Lopez et al., 2022). Impaired fibrotic regression occurs in aged mice after single BLM stimulation (Hecker et al., 2014; Qu et al., 2021; Selman & Pardo, 2014). To further confirm BTSA1 activity in vivo, we adopted single‐dose BLM instillation in both young and aged mice to establish reversible and irreversible lung fibrosis models respectively. We found that BTSA1 significantly reduced the number of senescent myofibroblasts in lung tissue and alleviated both reversible and irreversible pulmonary fibrosis.

In summary, our study demonstrates that high level of inactive BAX triggered by high levels of anti‐apoptosis agents in senescent myofibroblasts promote its apoptosis evasion, and a high level of inactivation of BAX‐mediated minority MOMP contributes to myofibroblast senescence by inducing DNA damage. BTSA1 protects mice from BLM‐induced pulmonary fibrosis by selectively eliminating senescent myofibroblasts via direct activation of BAX, and can be used as a novel senolytic drug for IPF for reversing established fibrosis.

## 4 MATERIALS AND METHONDS

### 4.1 Ethics statement

The ethics committee of the Central South University Science Research Center (Changsha, China) approved the experiments in this study, which were conducted in accordance with the guidelines of the National Institutes of Health. Mice were anesthetized with sodium pentobarbital (80 mg/kg, intraperitoneal injection) and every effort was made to minimize suffering before proceeding.

The animal study protocol was approved by the ethics committee of the Central South University Science Research Center (Changsha, China) (protocol code: 2020sydw0715).

### 4.2 Experimental animals and treatment

Eight‐week‐old and twenty‐month‐old male C57BL/6 mice (specific‐pathogen‐free [SPF] grade) were used in our experiments. All animals were maintained with free access to food and water. The mice were anesthetized with sodium pentobarbital (80 mg/kg, intraperitoneal injection) and every effort was made to minimize animal suffering. For reversible model (Zhang, Fu, et al., 2023), 8‐week‐old mice received an intratracheal instillation with BLM (50 μL, 1.25 mg/kg, Nippon Kayaku, Tokyo, Japan) at day 0, or with sterile PBS as a control. For irreversible model, 8‐week‐old mice received repetitive intranasal BLM instillation (20 μL, 1.00 mg/kg, Nippon Kayaku, Tokyo, Japan) once a week, or with sterile PBS as a control. And the 20‐month‐old were intratracheally injected with 50 μL of BLM (1.25 mg/kg, Nippon Kayaku, Tokyo, Japan). The aged mice were intraperitoneally injected with BTSA1 (10 mg/kg, Absin, China) on the 10th day after modeling, once every 2 days.

### 4.3 Histopathology evaluation

The lungs were isolated and fixed in 4% paraformaldehyde and embedded in paraffin. Hematoxylin and eosin staining, Masson's trichrome staining was used to assess tissue damage. Ashcroft fibrosis score was used for grading fibrosis scale and image J was used to calculate the collagen deposition area.

### 4.4 Fibroblast isolation and culture

Primary fibroblasts were isolated and purified from neonatal C57BL/6 mice aged 3–7 days (Zhou et al., 2019). In short, the lungs are separated under sterile conditions. The lung tissue was chopped and digested with collagenase I (1 mg/mL, Solarbio, Beijing, China) and DNase I (10 μg/mL, Roche, Germany) at 37°C for 1 h. Next, the cells were incubated at 37°C for several generations in DMEM/F‐12 (Procell, China) petri dishes containing 10% fetal bovine serum (FBS, Procell, China) and 1% penicillin–streptomycin mixture (Solarbio, Beijing, China). Cell passage to three to six generations.

For X‐ray irradiation, fibroblasts were cultured to the appropriate density, irradiated with 10Gy X‐rays, and cultured until 14 days for subsequent treatments, during which time the medium was replaced with fresh medium every 48–72 h.

For H_2_O_2_ stimulation, fibroblasts were subjected to H_2_O_2_ at 200 μm for 2 h per day for a total of 3 days, and incubation was continued for 48 h after the last H_2_O_2_ stimulation was completed.

For QVD‐OPH treatment, after receiving ionizing irradiation or H_2_O_2_ stimulation, primary lung fibroblasts were cultured with 30 μm QVD‐OPh and 15%FBS medium until harvest.

For BTSA1 treatment, after constructed the senescent model in vivo, the medium was replaced for 12 h with FBS‐free for cell starvation, and then treated 0.3 μm BTSA1 for 48 h.

### 4.5 Quantitative real‐time PCR

Total RNA was isolated from lung tissue and fibroblasts using trizol reagent (Takara, Kyoto, Japan) according to the manufacturer's protocol. Using A First Strand cDNA synthesis kit (Takara, Kyoto, Japan) for transcribing complementary DNA (cDNA) from 1 μg of total RNA. Real‐time PCR was performed using SYBR Green signal (Bio‐Rad, CA, USA) and Bio‐Rad real‐time PCR system (CFX96 Touch™, Bio‐Rad, USA). After normalizing the glyceraldehyde‐3‐phosphate dehydrogenase (GAPDH) gene or β2 microglobulin (B2M) by the formula 2^−ΔΔCT^, the relative mRNA expression was determined. The primer sequences are shown in Table S2.

### 4.6 Western blot analysis

Lung tissue and cells were prepared in cold RIPA lysis buffer (Bioss, Beijing, China) containing a proteinase inhibitor cocktail (Apexbio, Houston, TX, USA) for 30 min. Using a BCA kit (Thermo Scientific, MA, USA) to measure total protein concentration. Then, 20ug of total protein was separated by sodium dodecyl sulfate‐polyacrylamide gel electrophoresis (SDS‐PAGE) and transferred to polyvinylidene fluoride (PVDF) membranes (Millipore, Burlington, MA, USA), and blocking with 5% fat‐free milk for 1 h before incubated with the primary antibody overnight at 4°C. β‐tubulin or GAPDH was used as loading control. HRP‐conjugated anti‐mouse IgG (H + L) (1:5000; Signalway Antibody, College Park, MD, USA) and HRP‐conjugated anti‐rabbit IgG (1:5000; Signalway Antibody, College Park, MD, USA) were used as secondary antibodies for 1 h at room temperature (RT). Images were obtained using a ChemiDoc XRS system (Bio‐Rad) with enhanced chemiluminescence reagents (Millipore, MA, USA). The primary antibodies were anti‐α‐smooth muscle actin (α‐SMA) (42 kD, 1:1000; Servicebio, Wuhan, China), anti‐collagen I (Col 1) (130 kD, 1:1000; Proteintech, Wuhan, China), anti‐β‐tubulin (55 kD, 1:20000; Proteintech, Wuhan, China).

### 4.7 BAX conformational change assay

Measured the 6A7 epitope on activated BAX by immunoprecipitation assay in 20 mM Hepes pH 7.2, 150 mM KCl for 15 min at RT. The mixture (10%) was kept for input analysis, the rest of the mixture was mixed with 5 μL 6A7 antibody (sc‐23,959, Santa Cruz) and prewashed protein A/G beads (Santa Cruz) and incubated for overnight at 4°C with rotation. Washing with 1 mL of 3% BSA buffer 3 times, and then solubilized with 25 μL SDS loading buffer. Resolved by SDS‐PAGE electrophoresis and western blot analysis using anti‐BAX (20kD, 1:2000; Abcam, British).

### 4.8 BAX knock down

Primary fibroblasts were transfected with 50 nM siRNA targeting BAX (GCTCTGAACAGATCATGAA) or non‐targeting (from Guangzhou RIBO BIO., LTD) siRNA using Lipofectamine 3000 (Invitrogen) according to the manufacturer's instructions, after 72 h, these cells were exposed to X‐ray irradiation.

### 4.9 Immunohistochemistry

Fixed and embedded the mice lung tissue. Endogenous peroxidase activity was quenched with 0.3% H_2_O_2_ for 10 min, and the slides were blocked with 5% bovine serum albumin (BSA) for 30 min. Stained with primary antibodies α‐SMA (1:100; Servicebio, Wuhan, China), collagen I (1:100; Proteintech, Wuhan, China), collagen III (1:100; Proteintech, Wuhan, China), FN (1:50; Servicebio, Wuhan, China) overnight at 4°C, incubated with HRP‐conjugated secondary antibody (1:100; Sigma‐Aldrich, MI, USA) at RT for 1 h. Images were acquired on phase contrast microscope (Nikon, Tokyo, Japan).

### 4.10 Immunofluorescence staining

The lung sections were blocked with 5% BSA for 30 min and stained overnight at 4°C with rabbit anti‐p21 antibodies (1:500; Abcam, British), rabbit anti‐BAX (1:250; Abcam, British), mouse anti‐α‐SMA antibodies (1:200; Cell Signaling Technology, USA), mouse anti‐BAX(6A7) antibodies (1:50; Santa Cruz, USA), rabbit anti‐BCL‐XL (1:500; Abcam, British) overnight at 4°C, and then stained with Alexa Fluor 488‐goat anti‐rabbit secondary antibodies (1:200, Proteintech, Wuhan, China) and Alexa Fluor 594‐goat anti‐mouse secondary antibodies (1: 200, Proteintech, Wuhan, China) at RT for 1 h. In addition, 4′,6‐diamidino‐2‐phenylindole (DAPI; Proteintech, Wuhan, China) was used to stain the cell nuclei. Imaged on fluorescence microscope (Nikon, Tokyo, Japan).

The cells were fixed with 4% paraformaldehyde for 20 min and permeabilized with Triton X‐100 (0.1%, Solarbio, Beijing, China). Stained overnight at 4°C with rabbit anti‐p21 antibodies (1:500; Abcam, British), rabbit anti‐BAX (1:250; Abcam, British), mouse anti‐α‐SMA antibodies (1:200; Cell Signaling Technology, USA), rabbit anti‐BCL‐XL (1:500; Abcam, British), mouse anti‐BAX(6A7) antibodies (1:50; Santa Cruz, USA), rabbit anti‐γ‐H_2_AX (1:250; Abcam, British), rabbit anti‐H3K9me3 (1:400; Cell Signaling Technology, USA) overnight at 4°C, and then stained with Alexa Fluor 488‐goat anti‐rabbit secondary antibodies (1:200, Proteintech, Wuhan, China) and Alexa Fluor 488‐goat anti‐mouse secondary antibodies (1: 200, Proteintech, Wuhan, China) at RT for 1 h. In addition, 4′,6‐diamidino‐2‐phenylindole (DAPI; Proteintech, Wuhan, China) was used to stain the cell nuclei. Images were acquired on fluorescence microscope (Nikon, Tokyo, Japan).

### 4.11 SA‐β‐gal staining

Removed cell culture medium, washed with PBS for three times, fixed with β‐galactosidase fixing solution at RT for 15–20 min, cells washed again with PBS for three times, added the β‐galactosidase staining working solution and incubated in the 37°C, CO_2_‐free incubator overnight. Images were acquired on phase contrast microscope (Nikon, Tokyo, Japan).

### 4.12 Apoptosis

Cells were stained with annexin V and PI according to the manufacturer's instruction. Apoptotic cells were analyzed with FACS Caliber (Becton Dickinson, Heidelberg, Germany). Annexin V (+)/ PI (−) cells respond for apoptotic cells. Data were analyzed using the FlowJo 10.4.1 software (Tree Star, USA).

### 4.13 JC1 staining assays

Removed the cell culture medium, the cells were washed once with PBS, the cells were added with, incubated in 37° and 5%CO_2_ incubator with JC‐1 dyeing solution for 20 min, cells was washed for 2–3 times with JC‐1 buffer after incubation, and images were collected on phase contrast microscope (Nikon, Tokyo, Japan).

### 4.14 Caspase‐3 activity

Cells were treated with BTSA1, and caspase 3 activation was measured at the indicated time points by addition of the caspase 3 chemiluminescence reagent in accordance with the manufacturer's protocol (Beyotime, China). Luminescence was detected by a Luminoskan Microplate reader (Thermo).

### 4.15 Comet assay

Cells were collected suspended in PBS, embedded in 0.7% low melting agarose, uncoiled and electrophoretic under alkaline conditions, neutralized in neutral buffer, stained with propidium iodide solution, and imaged on phase contrast microscope (Nikon, Tokyo, Japan).

### 4.16 Statistical analysis

Data were presented as the mean ± SD. All experiments were repeated at least three times. The Student's *t*‐test compared means between the two groups. Comparisons between multiple groups were analyzed by one‐way analysis of variance followed by Student–Newman–Keuls (SNK) test. The threshold for statistical significance was set at *p* < 0.05. GraphPad Prism 8 was used for statistical analysis.

## AUTHOR CONTRIBUTIONS

*Conceptualization*: ZY and LZQ. Data curation: SMX, ZY, FJF. *Formal analysis*: SMX, ZY, FJF. *Investigation*: SMX, FJF, ZYN, CYF, LXH, CHP. *Project administration*: LZQ, ZY, SMX. *Resources*: LZQ, ZY, HY. *Software*: QYJ, SM. *Supervision*: LZQ, ZY. Validation: LZQ, ZY. *Writing—original draft*. SMX, ZY and *Writing—review & editing*: SMX, ZY, LZQ.

## FUNDING INFORMATION

This work was supported by grants from the National Natural Science Foundation of China (82070068, 82200085 and 82370077), the Natural Science Foundation of Hunan Province (2023JJ40805), the Natural Science Foundation of Changsha (kq2202116), and Central South University (1053320222199).

## CONFLICT OF INTEREST STATEMENT

The authors declare that they have no known competing financial interests or personal relationships that could have appeared to influence the work reported in this paper.

## Supporting information


Appendix S1.

Appendix S2.

Appendix S3.

## Data Availability

All data and materials used in the analysis are available to any researcher for purposes of reproducing or extending the analysis.
