# Supplementary material for: A novel senolytic drug for pulmonary fibrosis: BTSA1 targets apoptosis of senescent myofibroblasts by activating BAX
Source: Aging Cell. 2024 Jun 3;23(9):e14229. doi: 10.1111/acel.14229 (PMC11488301; doi:10.1111/acel.14229)
Supplement: Supplementary file 1 — Appendix S1. [file ACEL-23-e14229-s003.docx]

**Tables**

Table 1. Abbreviations

| α-SMA | α-smooth muscle actin |
| --- | --- |
| BCL-1 | A1 |
| BCL-2 | B-cell lymphoma |
| CCCP | Carbonyl cyanide m-chlorophenyl hydrazone |
| DCA | Deoxycholic acid |
| d.p.i. | Days post instillation |
| ECM | Extracellular matrix |
| H3K9Me3 | Tri-Methyl-Histone H3 (Lys9) |
| IPF | Idiopathic Pulmonary Fibrosis |
| IR | Irradiation |
| mΔψ | Mitochondrial membrane potential |
| MOMP | Mitochondrial outer membrane permeabilization |
| Minority MOMP | Minority mitochondrial outer membrane permeabilization |
| p16 | CDKN2A |
| p21 | CDKN1A |
| P53 | Tumor protein 53 |
| QVD-OPH | Quinolyl-valyl-O-methylaspartyl- [2,6-difluorophenoxy]-methyl ketone |
| ROS | Reactive oxygen species |
| SA-β-GAL | Senescence-associated-galactosidase |
| SASP | Senescence-associated secretory phenotype |

Table 2. Sequences of specific primers used in this study.

| Gene | Forward Primer Sequence (5 to 3) | Reverse Primer Sequence (5 to 3) |
| --- | --- | --- |
| *m-actin* | TTCCAGCCTTCCTTCTTG | GGAGCCAGAGCAGTAATC |
| *m-α-SMA* | CCAACTGGGACGACATGGAA | TCTGTCAGCAGTGTCGGATG |
| *m-Col 1* | CTGACGCATGGCCAAGAAGA | TACCTCGGGTTTCCACGTCT |
| *m-Col 3* | GTCTGGTGGCTTTTCACCCT | AGTTCGGGGTGGCAGAATTT |
| *m-CDKN2A* | CGCAGGTTCTTGGTCACTGT | TGTTCACGAAAGCCAGAGCG |
| *m-CDKN1A* | CCTGGTGATGTCCGACCTG | CCATGAGCGCATCGCAATC |
| *m-p53* | GCGTAAACGCTTCGAGATGTT | TTTTTATGGCGGGAAGTAGACTG |
| *m-MCP1* | TTAAAAACCTGGATCGGAACCAA | GCATTAGCTTCAGATTTACGGGT |
| *m-TGFβ* | TGCGCTTGCAGAGATTAAAA | CGTCAAAAGACAGCCACTCA |
| *m-IL6* | TAGTCCTTCCTACCCCAATTTCC | TTGGTCCTTAGCCACTCCTTC |
| *m-IL1β* | GCAACTGTTCCTGAACTCAACT | ATCTTTTGGGGTCCGTCAACT |
| *m-Ki67* | ATCATTGACCGCTCCTTTAGGT | GCTCGCCTTGATGGTTCCT |
| *m-PCNA* | TTTGAGGCACGCCTGATCC | GGAGACGTGAGACGAGTCCAT |
| *m-LAMB1* | GAAAGGAAGACCCGAAGAAAAGA | CCATAGGGCTAGGACACCAAA |
| *m-BAX* | TGAAGACAGGGGCCTTTTTG | AATTCGCCGGAGACACTCG |
| *m-BCL-XL* | GACAAGGAGATGCAGGTATTG | TCCCGTAGAGATCCACAAAAGT |
| *m-BCL2* | GTCGCTACCGTCGTGACTTC | CAGACATGCACCTACCCAGC |
