# Supplementary figures and images for: A novel senolytic drug for pulmonary fibrosis: BTSA1 targets apoptosis of senescent myofibroblasts by activating BAX
Source: Aging Cell. 2024 Jun 3;23(9):e14229. doi: 10.1111/acel.14229 (PMC11488301; doi:10.1111/acel.14229)

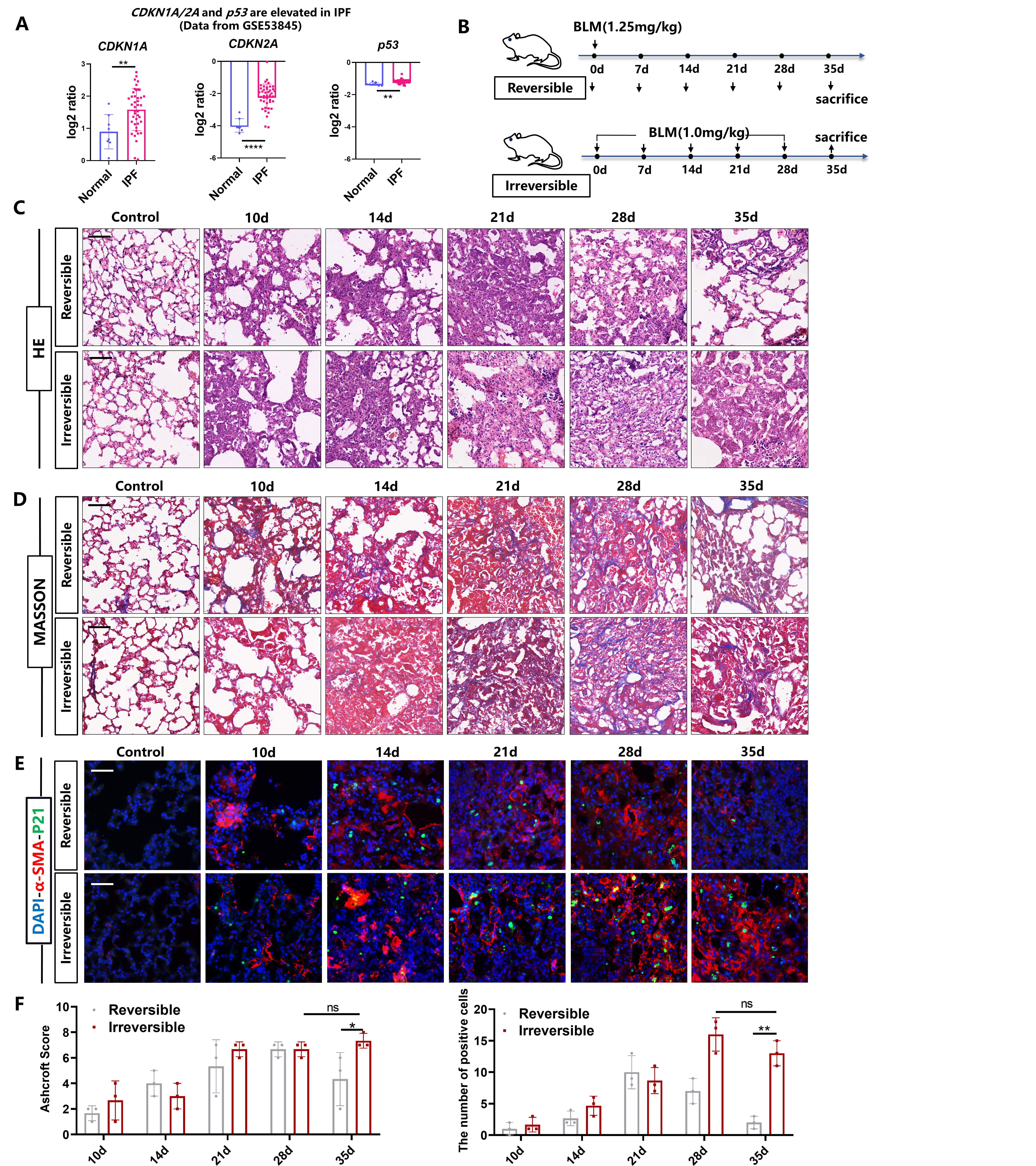

Supplement: Supplementary file 2 — Appendix S2. [file ACEL-23-e14229-s001.jpg]
