# Supplementary material for: A novel senolytic drug for pulmonary fibrosis: BTSA1 targets apoptosis of senescent myofibroblasts by activating BAX
Source: Aging Cell. 2024 Jun 3;23(9):e14229. doi: 10.1111/acel.14229 (PMC11488301; doi:10.1111/acel.14229)
Supplement: Supplementary file 3 — Appendix S3. [file ACEL-23-e14229-s002.docx]

Figure legend

Fig.S1 senescent cell accumulate in BLM-induced pulmonary fibrosis. (A) n = 42 patients with IPF; n = 8 normal controls. CDKN1A, CDKN2A, p53 increase in the IPF. Graph represents mean ± SEM (n=3); ***p*<0.01, *****p*<0.0001. (B) Schematic for single-dose BLM-induced reversible fibrosis model and repetitive BLM-injured irreversible model. (C) Representative micrographs of H&E of lung sections, scale bar =25 μm. (D) Representative micrographs of Masson staining of lung sections, scale bar =25 μm. (E) Immunofluorescent double-staining of α-SMA (Red) and p21 (Green) of lung sections of mice, scale bar = 50 μm. (F) Ashcroft score to grade fibrosis scale. The number of α-SMA^+^/p21^+^ cells in lung sections of mice. Graph represents mean ± SEM (n=3); **p*<0.05, ***p*<0.01, ns *p*>0.05.
